# Acute kidney injury in patients with idiopathic membranous nephropathy: influencing factors and prognosis

**DOI:** 10.1080/0886022X.2023.2194451

**Published:** 2023-03-30

**Authors:** Zhenzhou Li, Mengjie Weng, Lizhao Lin, Yi Chen, Jiaqun Lin, Jiong Cui, Dewen Jiang, Binbin Fu, Guifen Li, Caiming Chen, Yanfang Xu, Jianxin Wan

**Affiliations:** a Department of Nephrology, Blood Purification Research Center, The First Affiliated Hospital, Fujian Medical University, Fuzhou, China; b Fujian Clinical Research Center for Metabolic Chronic Kidney Disease, The First Affiliated Hospital, Fujian Medical University, Fuzhou, China; c Department of Nephrology, National Regional Medical Center, Binhai Campus of the First Affiliated Hospital, Fujian Medical University, Fuzhou, China

**Keywords:** Acute kidney injury, idiopathic membranous nephropathy, biopsy, prognosis

## Abstract

**Aim:**

Idiopathic membranous nephropathy (IMN) is a common type of nephrotic syndrome, and is associated with acute kidney injury (AKI). We investigated the association of multiple variables with AKI in patients with IMN.

**Methods:**

The data of 187 patients with biopsy-proven IMN were examined. Renal outcome was defined as progression to end-stage renal disease (ESRD). Binary logistic regression and Kaplan–Meier’s analysis were used for statistical analysis.

**Results:**

During follow-up, 46 (24.6%) patients developed AKI. The incidence of AKI was greater in males than females (*p* < .01). The AKI group had higher uric acid, lower serum PLA2R antibody positive, and worse baseline kidney function (all *p* < .01). Most patients in the AKI group had stage I (71.74%) or stage II (21.74%). The AKI group had higher renal tubular injury score and chronicity index (both *p* < .05). Binary logistic regression indicated that uric acid and baseline estimated glomerular filtration rate (eGFR) were independent risk factors for AKI in patients with IMN (*p* < .05). The optimal cutoff value of serum uric acid for predicting AKI was 402.50 μmol/L and the baseline eGFR was 96.83 mL/min/1.73 m^2^. Kaplan–Meier’s analysis showed that the cumulative renal survival rate was lower in the AKI group (*p* = .047).

**Conclusions:**

AKI increases the risk of poor prognosis in IMN patients and the high uric acid and low baseline eGFR were considered independent predictors for developing AKI in patients with IMN.

## Introduction

Idiopathic membranous nephropathy (IMN) is a common cause of primary nephrotic syndrome (PNS) in nondiabetic adults worldwide [1]. Xu et al. [2] analyzed the spectrum of glomerular diseases in China in 2016 and found that the proportion of patients with MN among all patients with glomerular diseases increased from 12.2% in 2004 to 24.9% in 2014. IMN is slowly progressive, prolonged, and often recurrent, and patient prognosis varies greatly. About one-third of patients experience spontaneous remission and some patients experience persistent urinary protein, but most patients have a chronic progressive disease or die from complications [3]. About 10–20% of IMN patients progress to end-stage renal disease (ESRD) [1].

Acute kidney injury (AKI) is a relatively common complication in patients with IMN, and patients who present with PNS are more likely to develop AKI. A recent study reported that AKI is the most serious complication of PNS, and patients who were elderly and had more underlying diseases had a poorer prognosis [4]. Failure to promptly identify and provide appropriate intervention for AKI in IMN patients increases the difficulty of follow-up treatment, morbidity and mortality, and the financial burden on patients. Therefore, it is important to identify risk factors for AKI in IMN so that appropriate interventions can be promptly applied.

The mechanism of secondary AKI in patients with PNS is not completely clear. It is believed that AKI in these patients may be related to intrarenal ischemia, renal interstitial edema, glomerular lesions, renal tubular necrosis, drug-related interstitial nephritis, and certain other conditions [5,6]. However, most recent studies have focused on AKI in patients with PNS [7–9], and little is known about AKI in patients with IMN. Our general purpose was to provide new information that improves the clinical management and care of patients with IMN. Therefore, we retrospectively analyzed the clinicopathological characteristics of IMN patients who presented at our hospital with or without AKI, the factors associated with AKI in these patients, and the factors associated with renal outcome in patients with IMN and AKI.

## Methods

### Patients and samples

This retrospective cohort study initially examined 208 patients with clinical manifestations of PNS who were diagnosed with MN based on renal biopsy and received follow-up for at least one year from September 2015 to August 2019 in the Department of Nephrology, The First Affiliated Hospital of Fujian Medical University. Patients were excluded if they had chronic renal insufficiency; secondary MN (SMN) due to systemic lupus erythematosus, hepatitis B viral infection, or allergic purpura; concomitant diseases, such as diabetic nephropathy, IgA nephropathy, focal segmental glomerular sclerosis (FSGS), proliferative glomerular nephritis, cryoglobulinemia, or tumor; or incomplete clinical data. This research was performed in accordance with the Declaration of Helsinki and was approved by the Ethics Committee of the First Affiliated Hospital of Fujian Medical University (MTCA, ECFAH of FMU [2015] 084-2). Informed written consent was obtained from all participants.

### Clinical and laboratory parameters

The clinical data of enrolled patients were collected at the time of renal biopsy, including sex, age, body mass index (BMI), systolic blood pressure (SBP), diastolic blood pressure (DBP), serum creatine (SCr), serum albumin, estimated glomerular filtration rate (eGFR), uric acid, total cholesterol (TC), triglycerides (TGs), serum phospholipase A2 receptor (PLA2R) status, 24 h urinary protein, urinary red blood cells, D-dimer, prothrombin time (PT), international normalized ratio (INR), use of a renin–angiotensin–aldosterone system (RAAS) inhibitor, use of diuretics, use of a glucocorticoid, and use of an immunosuppressant (including tacrolimus, cyclophosphamide, and cyclosporine).

The following five parameters were assessed during histopathology of the renal biopsy specimens: global sclerosis; glomerular segmental sclerosis; severity of tubulointerstitial changes; vascular lesions. Four semi-quantitative grades were used to classify serum PLA2R antibody titer: 0 (negative), 1+ (1:10), 2+ (1:32), 3+ (1:100), and 4+ (≥1:320). Tubulointerstitial changes, including tubular atrophy, interstitial fibrosis, and mononuclear cell infiltration, were expressed as a percentage of the area of cortical kidney tissue covered by lesions. These lesions were classified as none, mild (˂25%), moderate (25–50%), or severe (>50%). Semi-quantitative scores for the chronicity index (CI) and tubular interstitial lesion (TIL) were determined according to Austin et al. [10]. The intensity of the immunofluorescence staining of PLA2R and IgG4 glomerular deposits was graded using a semiquantitative scale (range: 0 to 4+), and a grade of 1+ or higher was considered positive.

### Follow-up and definition

Regular visits at intervals of 3 months were performed on every patient. Follow-up to March 2021, this study retrospectively analyzed the serum creatinine (SCr) level of each patient at the time of outpatient visit or readmission, and evaluated whether AKI or endpoint events occurred.

Baseline SCr level and baseline eGFR level were defined as SCr value and eGFR value that measured at the time of renal biopsy. The endpoint event in the present study was defined as ESRD, as indicated by an eGFR below 15 mL/min/1.73 m^2^ or the initiation of renal replacement therapy. AKI was diagnosed if any one of the following conditions were present during follow-up: an increase of SCr of at least 26.5 μmol/L within 48 h; an increase of SCr of at least 1.5 times above the baseline value within seven days; or hourly urine output below 0.5 mL/kg for more than 6 h. In addition, some patients had elevated SCr on the first day of admission; the baseline SCr values of these patients were assumed to be lower than on the first day based on the subsequent clinical course. Thus, based on the 2012 KDIGO Clinical Practice Guidelines for AKI [11], these patients were also diagnosed with AKI. Patients with AKI included in this study had been excluded from infection, heart failure, shock, trauma, poisonous plants, radiocontrast agents, NSAIDs use, post-renal and other secondary factors, and did not require dialysis treatment. AKI is staged for severity according to 2012 KDIGO Clinical Practice Guidelines for AKI [11]: AKI stage 1 was increase in SCr to 1.5–1.9 times baseline; AKI stage 2 was increase in SCr to 2.0–2.9 times baseline; AKI stage 3 was increase in SCr to 3.0 times baseline.

### Statistical analysis

All data were analyzed using SPSS version 25.0 (SPSS Inc., Chicago, IL). Continuous variables were presented as means ± standard deviations or medians with interquartile ranges (as appropriate). Differences between groups were assessed using the *t*-test or a nonparametric test (as appropriate) or with the Chi-square test (categorical variables). Survival curves for the AKI and non-AKI groups were generated using the Kaplan–Meier method, and were compared using the log-rank test. Binary logistic regression analysis and receiver operating characteristic (ROC) curve analysis were performed to evaluate the risk factors for AKI. A two-sided *p* value below .05 was considered statistically significant in all analyses.

## Results

### Patient characteristics

**Figure 1.**
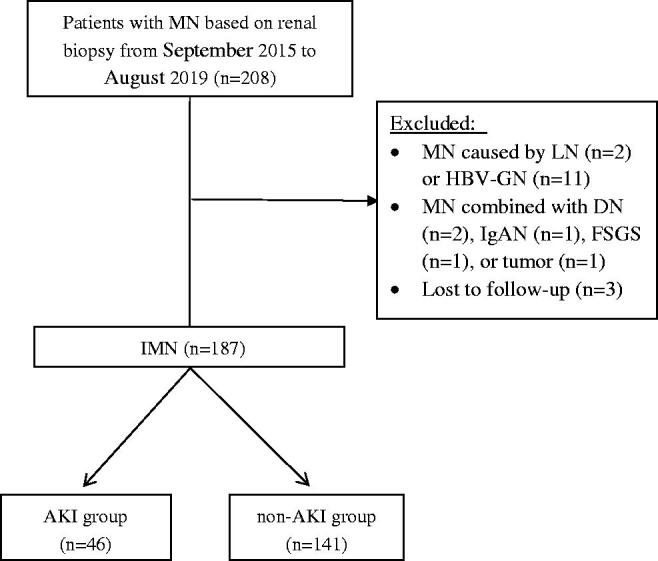
Disposition of study participants after initial recruitment. IMN: idiopathic membranous nephropathy; DN: diabetic nephropathy; IgAN: IgA nephropathy; FSGS: focal segmental glomerular sclerosis; AKI: acute kidney injury; LN: lupus nephritis; HBV-GN: hepatitis B virus associated glomerular nephritis.

We initially examined the records of 208 patients who were diagnosed with MN based on renal biopsy from September 2015 to August 2019. A total of 190 patients (91.3%) had IMN and 18 patients (8.7%) had SMN. Eleven of the patients with SMN (5.3%) had hepatitis B virus associated glomerular nephritis (Figure 1).

After excluding the patients with SMN and the three patients lost to follow-up, we included 187 IMN patients in the analysis. Overall, there were 130 males (69.52%) and 57 females (30.48%), corresponding to a male-to-female ratio of 2.28:1 (Table 1). The overall age range was 17–75 years, and the mean age was 53 years. The mean overall follow-up duration after admission was 37.5 ± 13.2 months, and the range was 18–62 months. During follow-up, a total of 46 patients (24.6%) developed AKI, of which 32 were stage 1 (69.57%), 11 were stage 2 (23.91%), and three were stage 3 (6.52%), and the other 141 patients (75.4%) were no AKI, and we excluded other factors that may impact AKI such as NSAIDs use, trauma, shock, etc., that may impact the rate of AKI. Patients were divided into the AKI group and the non-AKI group according to whether AKI had occurred or not. At the end of study, 10 patients (5.3%) overall progressed to ESRD.

**Table 1.** The characteristics of included IMN patients (*n* (%), ($\bar{x}\pm$*s*), *M*(*P*_25_, *P*_75_)).

	All (*n*= 187)	AKI (*n*= 46)	Non-AKI (*n*= 141)	*p* Value
Male sex (%)	130 (69.5)	40 (86.96)	90 (63.83)	.003*
Age (years)	53 (42,62)	55 (46,62)	52 (41,63)	.267
BMI (kg/m^2^)	23.74 (21.34, 25.78)	24.19 (21.58, 26.59)	23.64 (21.24, 25.63)	.157
SBP (mmHg)	133.32 ± 19.25	135.61 ± 18.38	132.57 ± 19.53	.354
DBP (mmHg)	80.99 ± 11.82	82.52 ± 13.05	80.49 ± 11.39	.312
Baseline SCr (μmol/L)	73.6 (56, 92)	93.5 (72.75, 110.93)	69 (54.05, 83.75)	<.001*
Serum albumin (g/L)	24.2 (20.8, 29.13)	22.95 (19.78, 29.33)	24.5 (21.4, 29.15)	.231
Baseline eGFR (mL/min/1.73 m^2^)	100.1 (75.7, 109.75)	81.05 (58.45, 100.65)	101.5 (89.8, 113.75)	<.001*
Uric acid (μmol/L)	385.99 ± 103.75	434.57 ± 122.71	369.92 ± 91.62	<.001*
TC (mmol/L)	7.78 ± 2.5	7.80 ± 2.34	7.76 ± 2.56	.923
TG (mmol/L)	1.97 (1.45, 2.67)	1.99 (1.54, 2.68)	1.97 (1.41, 2.68)	.896
PT (s)	10.8 (10.3, 11.5)	11 (10.30, 11.73)	10.80 (10.30, 11.50)	.439
INR	0.94 (0.9, 0.98)	0.95 (0.89, 1.01)	0.94 (0.90, 0.98)	.964
D-dimer (mg/mL)	0.79 (0.39, 1.72)	0.96 (0.57, 1.92)	0.70 (0.38, 1.655)	.107
Serum PLA2R antibody positive (%)	139 (74.3)	29 (63.0)	110 (78.0)	.044*
Serum PLA2R antibody (–/+/++/+++/++++)	48/15/35/65/24	17/0/9/13/7	31/15/26/52/17	.062
Hematuria (%)	104 (55.6)	28 (60.87)	76 (53.90)	.409
24 h urinary protein (g/d)	3.96 (2.38, 6.89)	4.54 (2.97, 7.79)	3.75 (2.18, 6.74)	.057
Therapy status				
RAAS inhibitor (%)	124 (66.3)	29 (63.04)	95 (67.38)	.589
Diuretics (%)	139 (74.33)	37 (80.43)	102 (72.34)	.275
Glucocorticoid (%)	81 (43.3)	25 (54.35)	56 (39.72)	.082
Immunosuppressant (%)	79 (42.2)	22 (47.83)	57 (40.43)	.378
Progression to ESRD (%)	10 (5.3)	5 (10.9)	5 (3.5)	.055

BMI: body mass index; SBP: systolic blood pressure; DBP: diastolic blood pressure; SCr: serum creatine; TC: total cholesterol; TG: triglyceride; PT: prothrombin time; INR: international normalized ratio; eGFR: estimated glomerular filtration rate; PLA2R: M-type phospholipase A2 receptor; RAAS: renin–angiotensin–aldosterone system; ESRD: end stage renal disease.

Data are expressed as numbers and percentages in non-continuous variables, as means ± standard deviation in parametric continuous variables, and as median and interquartile range in nonparametric continuous variables.

* Two-tailed *p*< .05.

### Comparisons of clinical characteristics

The AKI group had 40 males and six females, corresponding to a male-to-female ratio of 6.67:1, significantly higher than the ratio in the non-AKI group (1.76:1; *p* = .003, Table 1). The AKI group had significantly higher levels of baseline SCr and uric acid, and a significantly lower eGFR level and positive percentage of serum PLA2R antibody (all *p* < .05). However, the two groups had no significant differences in age, BMI, hematuria, 24-h urine protein, serum TC, serum TGs, serum albumin, PT, D-dimer, INR, serum PLA2R antibody grade, and use of different therapies (all *p* > .05).

### Comparisons of pathological features

We examined the pathological stage and other features of all patients based on renal biopsy results (Table 2). Overall, most patients were in stage I (*n* = 65.78%), followed by stage II (*n* = 58, 31.02%), stage III (*n* = 5, 2.67%), and stage IV (*n* = 1, 0.53%). Most patients in the AKI group were in stage I (*n* = 33, 71.74%) and stage II (*n* = 10, 21.74%), but the AKI and non-AKI groups did not significantly differ in pathological stages (*p* > .05). The TIL and CI were significantly higher in the AKI group (both *p* < .05). However, the two groups had no statistically significant differences in glomerulosclerosis, mesangial proliferation, tubular atrophy, interstitial inflammatory infiltration, interstitial fibrosis, crescent formation, renal microangiopathy, kidney IgG4 deposition, and kidney PLA2R staining (all *p* > .05).

**Table 2.** The pathological features of included IMN patients (*n* (%), *M*(*P*_25_, *P*_75_)).

	All (*n* = 187)	AKI (*n* = 46)	Non-AKI (*n* = 141)	*p* Value
Pathological stage				
Stage I (%)	123 (65.78)	33 (71.74)	90 (63.83)	.126
Stage II (%)	58 (31.02)	10 (21.74)	48 (34.04)	
Stage III (%)	5 (2.67)	2 (4.35)	3 (2.13)	
Stage IV (%)	1 (0.53)	1 (2.17)	0	
Glomerular sclerosis (%)	75 (40.10)	24 (52.17)	51 (36.17)	.054
Mesangial proliferation (%)				
No proliferation	138 (73.80)	34 (73.91)	104 (73.76)	.902
<25%	39 (20.86)	9 (19.57)	30 (21.28)	
>25%	10 (5.34)	3 (6.52)	7 (4.96)	
Tubular atrophy (%)				
No atrophy	10 (5.34)	1 (2.17)	9 (6.38)	.051
<25%	151 (80.75)	34 (73.91)	117 (82.98)	
25–50%	25 (13.37)	10 (21.74)	15 (10.64)	
>50%	1 (0.53)	1 (2.17)	0	
Interstitial inflammatory infiltration (%)				
No infiltration	4 (2.14)	0	4 (2.84)	.137
<25%	160 (85.56)	37 (80.43)	123 (87.23)	
25–50%	20 (10.70)	7 (15.22)	13 (9.22)	
>50%	3 (1.60)	2 (4.35)	1 (0.71)	
Interstitial fibrosis (%)				
No fibrosis	14 (7.49)	2 (4.35)	12 (8.51)	.273
<25%	153 (81.82)	37 (80.43)	116 (82.27)	
25–50%	17 (9.09)	5 (10.87)	12 (8.51)	
>50%	3 (1.60)	2 (4.35)	1 (0.701)	
Crescent formation (%)	7 (3.74)	1 (2.17)	6 (4.26)	.518
Renal microangiopathy (%)	25 (13.37)	8 (17.39)	17 (12.06)	.356
TIL	3 (3, 3)	3 (3, 3.25)	3 (3, 3)	.021*
CI	2 (2, 3)	3 (2, 3.25)	2 (2, 3)	.034*
Kidney IgG4 deposition (%)	143 (76.47)	34 (87.18)	109 (89.34)	.709
Kidney PLA2R staining (%)	128 (68.45)	28 (71.79)	100 (81.97)	.171

TIL: tubular interstitial lesion; CI: chronic index; PLA2R: M-type phospholipase A2 receptor.

Data are expressed as numbers and percentages in non-continuous variables and as median and interquartile range in nonparametric continuous variables.

* Two-tailed *p*< .05.

### Risk factors for concomitant AKI in IMN patients

**Figure 2.**
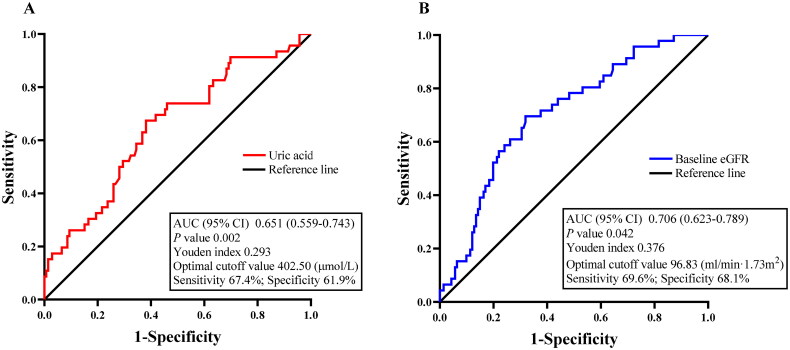
ROC curve analysis of the predictive performances of (A) serum uric acid and (B) baseline eGFR levels for AKI.

Based on the above univariate analysis, we considered gender, baseline eGFR, uric acid, TIL, and CI as factors possibly influencing AKI in IMN patients. Thus, we performed binary logistic regression analysis that controlled for confounding by these five factors. The results showed that uric acid (*p* = .006) and baseline eGFR (*p* = .003) were independent risk factors for AKI in these IMN patients (Table 3). Next, we generated ROC curves to determine the predictive value of serum uric acid and baseline eGFR levels for AKI (Figure 2). The area under the curve (AUC) value of serum uric acid was 0.651, the optimal cutoff (based on Youden’s index) for predicting AKI was 402.50 μmol/L, the sensitivity was 67.4%, and the specificity was 61.9% (Figure 2(A)). In addition, the AUC value representing the predictive value of the baseline eGFR was 0.697 and the optimal cutoff value was 96.83 mL/min/1.73 m^2^ with high specificity (68.1%) and sensitivity (69.6%) (Figure 2(B)). These results imply that serum uric acid and baseline eGFR levels have potential values for the prediction of AKI in patients with IMN.

**Table 3.** Multifactorial analysis of IMN patients with AKI (logistic).

	*B*	Wald	HR (95%CI)	*p* Value
Male sex	0.926	3.271	2.524 (0.925–6.883)	.071
Baseline eGFR	–0.023	8.680	0.978 (0.963–0.992)	.003*
Uric acid	0.005	7.410	1.005 (1.001–1.009)	.006*
TIL	0.087	0.284	0.916 (0.525–1.599)	.758
CI	0.152	0.199	1.165 (0.597–2.273)	.655

HR: hazard ratio; 95% CI: 95% confidence interval; eGFR: estimated glomerular filtration rate; PLA2R: M-type phospholipase A2 receptor; TIL: tubular interstitial lesion; CI: chronic index.

* Two-tailed *p*< .05.

### Long-term renal outcome of IMN patients with AKI

**Figure 3.**
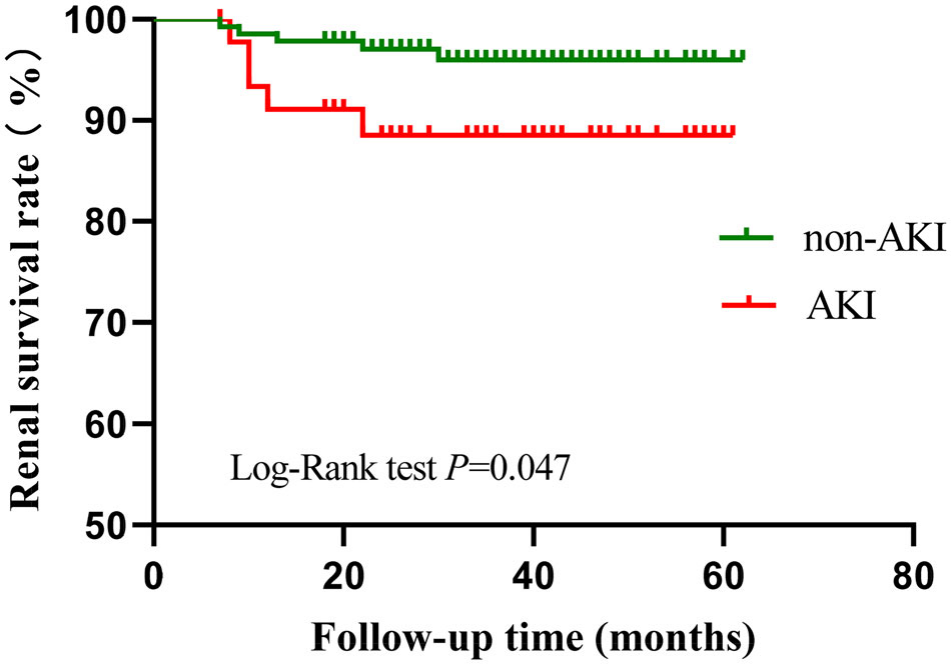
Kaplan–Meier’s analysis of renal survival of IMN patients in the AKI group and the non-AKI group.

At the end of follow-up, five (10.9%) patients in AKI group and five (3.5%) patients in non-AKI group reached renal endpoint events, separately. Kaplan–Meier’s analysis of renal survival in the AKI and non-AKI groups showed that cumulative renal survival was significantly lower in the AKI group (log-rank test: *p* = .047; Figure 3).

## Discussion

IMN is a common pathological cause of PNS, and these patients are generally sensitive to glucocorticoid therapy. IMN patients usually present with hypertension, lower extremity edema, hematuria, and renal dysfunction [12], and certain patients have an increased risk of developing AKI. Although most IMN patients recover and regain normal renal function after AKI, some develop chronic renal insufficiency or even require long-term renal replacement therapy. Previous studies showed that IMN increased the risk of secondary AKI, but the reasons for this and the underlying mechanisms are uncertain. The occurrence of AKI in IMN patients increases the length of hospitalization, hospitalization costs, in-hospital mortality, and the time needed for remission [13]. These patients also experience increased long-term adverse outcomes, such as accelerated progression of chronic kidney disease (CKD), development of ESRD, and increased risk of cardiovascular events [14,15]. Therefore, early detection, appropriate preventive measures, and active treatment are all needed to improve the outcomes of these patients.

We found that the incidence rate of AKI in our IMN patients was 24.6% during follow-up. By comparison, two recent studies [8,16] of PNS showed that adults with mild glomerular disease were most susceptible to AKI, and the incidence rate of AKI in these patients was between 24.11% and 38.42%, which was consistent with our results.

Our results showed that patients in the AKI group had significantly higher TIL and CI scores than the non-AKI group. Smith and Hayslett [17] studied a series of cases who had severe AKI with PNS and found that 60% of them had abnormalities in the tubular interstitial compartment that were suggestive of acute tubular necrosis. Other studies [18,19] showed that pathologic changes, including chronic inflammation, vascular dysfunction, and interstitial tubular fibrosis, may contribute to the increased vulnerability of patients with AKI. Most renal biopsies in our patients were not performed at the time of the AKI episode, which made it difficult to identify the relationship of specific pathological changes with the development of AKI. However, our analysis of baseline renal biopsies in IMN patients suggested that patients with high TIL and CI scores may be associated with AKI.

A meta-analysis showed that lower eGFR is associated with higher risks of AKI among individuals with or without either diabetes or hypertension [20]. Recent evidence suggests that reduced baseline eGFR is significantly associated with increased odds of AKI in hospitalized COVID-19 patients [21]. Similarly, our study suggested that lower baseline eGFR is associated with higher risks of AKI in IMN patients, even after adjustment for gender, uric acid, TIL, and CI.

In addition, we found that IMN patients with high levels of serum uric acid had an increased risk for AKI, in agreement with previous research [22,23]. Other research found that hyperuricemia increased the risk of AKI in patients who had PNS [24,25]. This is likely because an elevated serum uric acid level activates the RAAS, disrupts hemodynamics, and leads to renal vasoconstriction, endothelial dysfunction, and renal interstitial damage [26]. In addition, a sharp increase in serum uric acid can lead to the formation of uric acid crystals in the renal tubules, followed by blockage of the renal tubules or compression of the distal renal blood vessels, all of which can lead to AKI [27]. A previous study of an animal model in which cisplatin was used to induce AKI showed that a high uric acid level led to increased renal inflammation with further aggravation of kidney damage, and treatments that reduced the serum uric acid level reversed this damage [28]. Therefore, based on our logistic regression analysis and the results of these previous studies, hyperuricemia may be an early indicator of AKI in patients with IMN, and based on the ROC curve, the optimal cutoff for predicting AKI was 402.50 μmol/L. It could therefore be useful as an indicator of the need for early intervention.

Many studies showed that patients who had AKI with an underlying disease accelerated progression to ESRD [29,30]. In agreement, we also found that the cumulative renal survival rate was significantly lower in our AKI group, which suggested that the occurrence of AKI increases the risk of IMN patients developing ESRD. A clinical study of IMN patients [5] reported that the cumulative kidney survival rate in AKI patients was 67.1% (±5.3) at 2 years and 43.7% (±7.3) at 4 years; these numbers are significantly lower than in our non-AKI patients (99.5%±0.5 at 2 years and 92.5%±4.2 at 4 years). This previous study also reported the complete remission rate was obviously lower in AKI patients than in non-AKI patients [5]. Thus, AKI should not be neglected, but instead should be considered an independent predictor of mortality and an important contributor to CKD.

There are several limitations to the present study. First, we excluded patients who likely had IMN but did not receive renal biopsies, and this group presumably had a lower incidence of AKI than the patients we studied. Second, our study was in lack of contemporaneous non-AKI control, pathological and therapeutic change data during the course of AKI development meant that we could not infer causal relationships with a dynamic perspective. Third, more detailed subgroup analysis could not be performed on the prognosis of IMN patients with different recovery conditions after AKI due to the insufficient sample size. In addition, all patients were from a single center in China. Therefore, a prospective multi-center study with a large sample is required to validate our conclusions.

## Conclusions

In summary, we found that the incidence of AKI in IMN patients was 24.05% during follow-up and IMN patients with AKI were more likely to progress to ESRD. Our results also indicated that the severity of serum uric acid and baseline eGFR could be considered independent predictors for developing AKI in patients with IMN. Therefore, when examining patients with IMN, we suggest inclusion of serum uric acid and baseline eGFR in risk scoring systems that are used to predict AKI.

## Supplementary Material

Supplemental Material
