# Supplementary figures and images for: Acute kidney injury in patients with idiopathic membranous nephropathy: influencing factors and prognosis
Source: Ren Fail. 2023 Mar 30;45(1):2194451. doi: 10.1080/0886022X.2023.2194451 (PMC10064812; doi:10.1080/0886022X.2023.2194451)

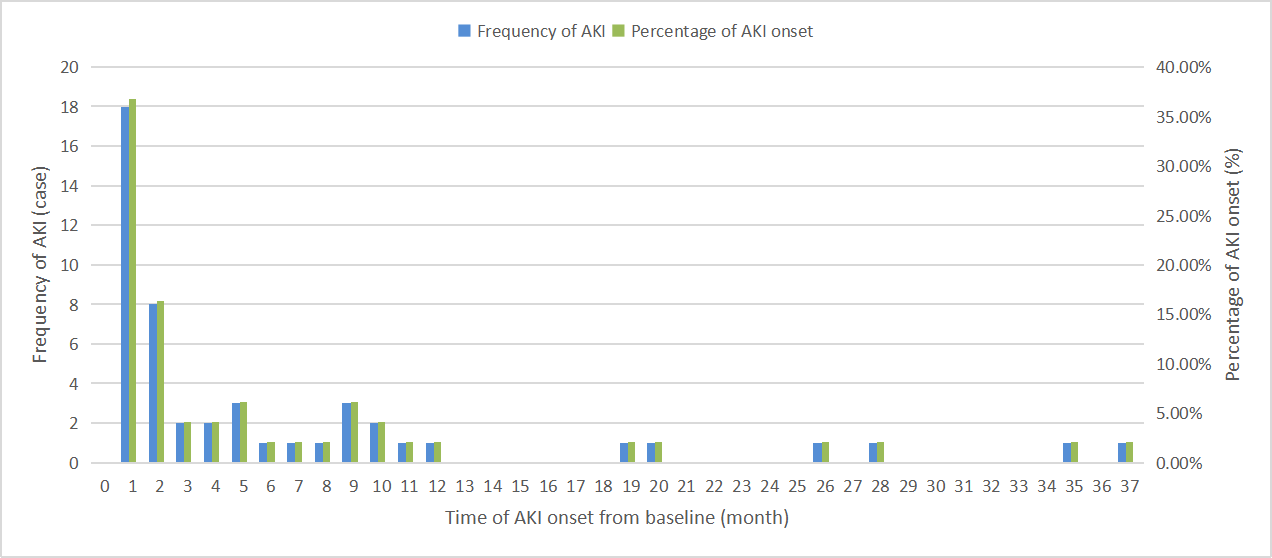

Supplement: Supplemental Material [file IRNF_A_2194451_SM3039.png]
